# Couple therapy in the 2020s: Current status and emerging developments

**DOI:** 10.1111/famp.12824

**Published:** 2022-09-29

**Authors:** Jay Lebow, Douglas K. Snyder

**Affiliations:** ^1^ Family Institute of Northwestern Northwestern University Evanston Illinois USA; ^2^ Department of Psychological and Brain Sciences Texas A&M University College Station Texas USA

**Keywords:** clinical practice, couple therapy, marital therapy, psychotherapy, relationships

## Abstract

This paper provides a critical analysis and synthesis of the current status and emerging developments in contemporary couple therapy. Its narrative centers on the evolution of couple therapy into a prominent intervention modality and coherent body of practice. The review begins with the consideration of the field's strong empirical underpinnings derived from research on couple therapy and basic relational science. Couple therapy comprises the widely accepted method for reducing relationship distress and enhancing relationship quality. Moreover, both as a stand‐alone intervention and in conjunction with other treatment formats, couple‐based interventions have garnered considerable empirical support for their effectiveness in addressing a broad spectrum of specific relational dysfunctions as well as individual emotional and physical health problems. We highlight the convergence of methods through common factors, shared strategies, and remarkably similar arrangements across approaches. Our review also points to key differences among approaches, the importance of recognizing respective strengths and limitations linked to these differences, and building on differences across models when selecting and tailoring interventions for a given couple. The discussion concludes with a consideration of recent trends in the field including the impact of telehealth and related digital technologies, the expansion of specific treatments for specific problems and diverse populations, the interface of couple therapy with relationship education, and enduring challenges as well as new opportunities addressing broader systemic and global dynamics.

## Abstract (zh)

本文对当代伴侣治疗的现状和新发展进行了批判性地分析和综合。整个叙述集中在伴侣治疗演变成一个突出的干预模式和连贯的实践体系。该综述首先考虑了该领域从伴侣治疗和基础关系科学的研究中获得的强有力的经验基础。伴侣治疗包括被广泛接受的减少伴侣关系困扰和提高伴侣关系质量的方法。此外，以伴侣为基础的干预，无论是作为独立的干预手段还是与其他治疗形式相结合，在解决各种各样的特定关系功能障碍以及个人情感和身体健康问题方面的有效性，都获得了相当多的经验支持。我们强调实现各种方法的融合，主要通过共同因素、共享策略和不同方法之间非常相似的组合安排来实现。本综述研究还指出了不同方法之间的关键的差异点，认识到与这些差异相关的各自优势和局限性的重要性，以及在为特定的伴侣选择和定制干预措施时建立在不同模式的差异上。讨论最后考虑了该领域最近的发展趋势，包括远程医疗和相关数字技术的影响，针对特定问题和不同人群的特定治疗的扩展，伴侣治疗与关系教育的衔接，以及持久的挑战和解决更广泛的系统和全球动态的新机遇。.

## INTRODUCTION

This paper is occasioned by our completing the editing of a major handbook of couple therapy (Lebow & Snyder, 2023). In experiencing the breadth of the field of couple therapy over the 4 years of preparing that book, we noticed emerging trends in the field, shared visions, differences among approaches, and exciting recent developments. Here, we summarize what is in part simply a “sifting of the data” from what we read but also inevitably our own effort to make sense of the common ground and diversity in couple therapy. We look to extrapolate from the vast array of writing and presentations about couple therapy, broad trends in the field, as well as commonalities and continuing major points of difference and controversy across approaches. So, what then can we say of couple therapy?

## THE PROMINENCE OF COUPLE THERAPY

Couple therapy has emerged as an important, widely disseminated form of therapy. Although there was a time when couple therapy was mostly an afterthought in considerations of psychotherapy and counseling, primarily consisting of methods derived from individual or family therapy and adapted to couples, couple therapy has evolved into a form of treatment that stands on its own, is widely practiced, and has its own distinct methods. The largest international study of psychotherapists found that 70% of psychotherapists treat couples (Orlinsky & Ronnestad, 2005). A survey of expert psychotherapists' predictions about future practices in psychotherapy showed couple therapy to be the format likely to achieve the most growth in the next decade (Norcross et al., 2013) and this projection appears to have been confirmed.

Three key factors have driven the development and widespread adoption of couple therapy as a prominent therapeutic modality. The first is the high prevalence of couple distress. In the United States, 40%–50% of first marriages end in divorce (Kreider & Ellis, 2011). Globally, across almost all countries for which data are available, divorce rates increased from the 1970s to the beginning of this century (Organization of Economic Cooperation and Development, 2011) and divorce has become commonplace even in countries where it once was rarely encountered (Doherty et al., 2021). Even for those less at risk for divorce, many couple relationships experience periods of significant turmoil.

The second factor prompting the rising profile of this set of methods is the adverse impact of relationship distress on the emotional and physical well‐being of adult partners and their offspring. In a survey in the United States, the most frequently cited causes of acute emotional distress were couple relationship problems (Swindle et al., 2000). Partners in distressed relationships are significantly more likely to have a mood disorder, anxiety disorder, or substance use disorder (McShall & Johnson, 2015) and to develop more physical health problems (Waite & Gallagher, 2000). Moreover, couple distress has been related to a wide range of deleterious effects on children, including mental and physical health problems, poor academic performance, and a variety of other concerns (Bernet et al., 2016).

A third factor propelling the prominence of couple therapy is the evolution of higher expectations for relationship life. Whereas once relational misery was simply to be tolerated, today couples have much higher expectations of relational life and see couple therapy as the pathway to better relationships (Cherlin, 2009; Dowbiggin, 2014; Finkel, 2017).

## COUPLE THERAPY: AN EVOLVING FIELD

Couple therapy is a constantly evolving field. Principles of couple therapy have emerged that transcend theoretical orientation, as have several widely disseminated specific approaches to couple therapy aimed at reducing couple distress and improving relationship quality. Still, other couple‐based interventions have been developed targeting specific couple or individual problems (e.g., partner aggression, infidelity, and depression) and populations (e.g., emerging adults, LGBTQ couples, and stepfamily couples). Although there remain threads of both theoretical and technical connection to various methods of individual and family therapy (Lebow, 2014), the field now includes a distinct set of prominent approaches, builds on an enormous body of basic research focused on intimate relationships, and offers a substantial body of empirical evidence supporting the efficacy and effectiveness of its methods. Thus, it has become abundantly clear that effective intervention with couples requires its own set of theories, approaches, and methods anchored in relational science.

### A brief history of couple therapy

In their classic overview of couple therapy, Gurman and Fraenkel (2002) described stages in the development of the field. First, in the early 20th century, atheoretical marriage counseling began to be practiced, featuring a pragmatic mix of psychoeducation and advice giving. During this stage, most of those working with couples did not label themselves as psychotherapists, and often they did not see spouses together. The second stage of the field, psychoanalytic experimentation, began in the 1930s, expanding from the then predominant form of therapy, psychoanalytic psychotherapy, to work with couples. Mostly, partners tended to be seen separately in this treatment by the same therapist in what has come to be called concurrent therapy, though eventually, this work segued into the beginnings of conjoint therapies in which both spouses participated in sessions with a therapist. Nonetheless, Michaelson (1963) estimated that in the 1940s, only 5% of couples were seen conjointly, and by the mid‐1960s, this number had only increased to about 15%. The third phase of couple therapy stemmed from the cataclysmic impact of the family therapy revolution in the 1960s and 1970s, in which several prominent models of systemic therapy emerged sharing the common ground of being highly influenced by systems theory. Subvariations of such core family systems therapies as experiential, strategic, structural, psychoanalytic, intergenerational, and behavioral therapies focused on couples and couple therapy (Gurman & Kniskern, 1981). In these therapies with their interactional basis, partners were almost always seen conjointly.

Through a different lens, couple therapy also evolved in relation to sociocultural influences. Dowbiggin (2014) described a historical shift in couples' looking for guidance primarily from family and community to their seeking help from counseling professionals. He also suggested that marriage counseling—with its emphasis on personal happiness, sexual satisfaction, and more modern gender roles—both fit within and contributed to the cultural context of middle‐class 20th‐century America. Doherty (2020) similarly situated the development of couple therapy in the context of 20th‐century family life, subject to larger system factors such as the rise in the divorce rate, the emergence of feminism, the explication of multicultural perspectives, and changes in American culture's view of marriage.

In the most recent phase of couple therapy in the 21st century, couple therapy has emerged as a mature discipline. Couple therapy has come to incorporate a wide array of distinct treatments, and a stronger evidence base both in the efficacy of therapies and in its foundation in the emerging body of relational science. Couple therapy has also broadened its conceptual framework to incorporate feminism, multiculturalism, and a broader view of gender and sexuality. Thus, “couple” now speaks to a much broader diversity of couples, and with this change has come an update from labeling “marital therapy” to “couple therapy.” Indeed, the continuing evolution of couple therapy now incorporates the increased use of social media and technology as well as open discussions about LGBTQ rights, gender equity, racism, social justice, politics, sexuality, individuality, freedom, and gender identity (Doherty, 2020). This era also includes the flourishing of numerous integrative methods and the development of couple therapy as a format for treating problems of individual partners.

## COUPLE THERAPY WORKS!

Reviews and meta‐analyses affirm the effectiveness of couple therapy in reducing relationship distress (Bradbury & Bodenmann, 2020; Doss et al., 2022; Lebow et al., 2012; Roddy et al., 2020; Shadish & Baldwin, 2003). Cognitive‐behavioral couple therapy, integrative behavioral couple therapy, and emotionally focused couple therapy each have sufficient evidence to be considered specific well‐established treatments for relationship distress. Nonetheless, broadly, meta‐analyses show behavioral and nonbehavioral therapies to have similar rates of impact (Shadish & Baldwin, 2005). The average person receiving couple therapy is better off at termination than 70%–80% of individuals not receiving treatment—an improvement rate that rivals or exceeds the most effective psychosocial and pharmacological interventions for individual mental health disorders. A variety of couple treatments have also garnered evidence supporting their effectiveness for specific relationship problems including sexual difficulties (McCarthy & Thestrup, 2008), infidelity (Baucom et al., 2006), and intimate partner violence (Epstein et al., 2015; Stith et al., 2011). Yet, there is some indication that effectiveness in clinical settings in which treatments are not closely monitored is somewhat lower than in controlled trials (Bradbury & Bodenmann, 2020). Further, there is evidence that as with many problems, the impact of most couple therapies dissipates for about half the couples over several years of follow‐up (Bradbury & Bodenmann, 2020).

In addition to reducing either general or specific relationship difficulties, evidence from several clinical trials supports the beneficial impact of couple therapies for coexisting emotional, behavioral, and physical health concerns (Babinski & Sibley, 2022; Fischer et al., 2016; Goger & Weersing, 2022; Hogue et al., 2022; Lamson et al., 2022; Stith et al., 2022). For example, there is evidence in support of couple‐based interventions for depression or anxiety (Wittenborn et al., 2022), posttraumatic stress (Monson et al., 2012), and alcohol problems (McCrady et al., 2016) of an adult partner. Couple‐based interventions for physical health problems comprise an expanding application—with evidence beginning to emerge supporting the benefits of couple therapy across a broad spectrum of conditions including couples in whom one partner has cancer, chronic pain, cardiovascular disease, anorexia nervosa, or type‐2 diabetes (Fischer et al., 2016; Lamson et al., 2022; Rohrbaugh et al., 2012; Shields et al., 2012; Woods et al., 2020). Typical components of couple‐based interventions for individual mental and physical health problems emphasize partner support, improved communication, and increased attention to the disorder's adverse impact on the couple relationship. The extension of couple‐based treatments to individual disorders reflects one of the most important developments of couple therapy in this century.

## FOUNDATION IN RELATIONAL SCIENCE

An important aspect of contemporary couple therapy is its strong foundation in relational science. Consider that couple therapy began as a method of practice before there was a field of relational science. Indeed, at the time of its origin, there were only the most primitive beginnings of social psychology. The infusion of relational science into practice has been steady and evolving.

The first widely recognized connections to science came in the form of bringing outcome and efficacy assessments to couple therapies (Gurman & Kniskern, 1981). To no great surprise, those efforts initially instigated considerable reactivity from those who eschewed a focus on measurable outcomes and who practiced therapies less frequently represented in the research literature (Gurman & Kniskern, 1978). In historical context, it is ironic that Alan Gurman, who espoused a nuanced view of the therapy process and outcome, was the primary mover of this initial emphasis on outcomes (Gurman & Kniskern, 1978), yet even his nuanced view led to a strong negative reaction. Today, the crucial role of evidence in relation to the impact of various couple therapies is widely accepted. Most couple therapy begins with the clear purpose of reducing relationship distress and promoting couple well‐being, measurable outcomes that readily can be compared to the limited changes in relational satisfaction typical of those couples in no‐treatment control conditions (Baucom et al., 2003; Roddy et al., 2020).

To some extent, couple therapy has become more firmly established because both meta‐analytic data and systematic reviews of the literature affirm the considerable broad impact of couple therapy (Bradbury & Bodenmann, 2020; Doss et al., 2022; Roddy et al., 2020; Shadish & Baldwin, 2003, 2005) and of several of its specific approaches (Fischer et al., 2016; Roddy et al., 2016; Wiebe & Johnson, 2016). Research also highlights the impact of couple therapy on individual functioning even when relational functioning is the primary focus of couple therapy. Moreover, unlike the spontaneous remission of some problems that occur in the absence of treatment, research demonstrates little improvement in relationship satisfaction among distressed couples who do not receive therapy (Baucom et al., 2003; Roddy et al., 2020). Mental health and other healthcare delivery systems find links of couple‐based treatments to such clear and measurable outcomes essential.

Even more marked has been the influence of basic relational science research on couple therapy. Whereas the early forms of couple therapy only drew occasionally on the emerging field of relational science, most approaches now cite basic research about relationships as part of the foundation for their methods. Included here are such threads as research about attachment, communication processes, behavior exchanges, and emotional resonance, as well as characteristics of couples with specific problems or from specific populations. The linkages between basic research and practice articulated by Gottman (1999) in the late 20th century modeled for others the incorporation of such basic science research into practice. After the emergence of science‐based couple therapies, those who promoted their ideas about relationships without spelling out the empirical basis of those concepts and methods came to have less credibility (even if remaining fashionable at times in the popular media). Moreover, with the empirical investigation also came the ability to disconfirm theories and even identify the potentially harmful effects of certain untested ideas.

## LINKS TO NEUROSCIENCE

Closely connected to the incorporation of relational science in practice has been the rapid advance in the last decade in the integration of relational neuroscience into contemporary approaches. Most models of couple therapy developed before the technology was available to assess brain function in relational life. Nonetheless, with the explosion in the information available from neuroscience in relation to couple functioning, couple therapies have begun to incorporate this emerging and exciting new knowledge base. Most especially, Fishbane's (2015) translation of neurobiology to the couple context has had considerable influence, providing a bridge to couple therapists directly being able to invoke working with neural pathways as a part of their repertoire. Other applications of neuroscience have become an essential part of emotionally focused couple therapy (Greenman et al., 2019) and Gottman method therapy (Gottman & Gottman, 2015) as well as many other specific approaches (Tatkin, 2011).

Yet, here there is a caveat. Relational neuroscience is in its infancy. Studies are complex with endless possible neurotransmitters and brain structures that may be simultaneously influencing and influenced by couple processes. Methodologies range from those employing simple, readily available instruments such as pulse oximeters (an inexpensive instrument that many bought to monitor the effects of Covid‐19 that has utility here) to very expensive fMRI scanners. In exploring the literature and evaluating claims made of the implications of findings for clinical practice, it is vital to understand that specific findings that support one approach might also support another, that some findings come from a single study yet all research findings require replication and testing across diverse contexts before they can be seen as broadly applicable, that sometimes claims are made that inappropriately extend correlations to infer causation, and that the body of findings from neuroscience is only just beginning to produce an evidence‐based set of knowledge that is widely accepted.

## CONVERGING METHODS

One of the most prominent trends in couple therapy is an emerging and substantial convergence of specific intervention methods across different theoretical approaches.

### Couple therapy is both pluralistic and integrative

Contemporary couple therapies often cross the boundaries of schools of therapy and theoretical constructs that typically have been identified in individual therapy and earlier iterations of couple therapy. Thus, for example, cognitive‐behavioral couple approaches today transcend simply focusing on cognitions and behavioral sequences, instead also tapping emotion, meaning, and early experience (Baucom et al., 2019; Epstein et al., 2016). Similarly, while psychoanalytic individual therapy almost exclusively focuses on such factors as transference, the impact of early experience, and inner experience, the couple therapy variations of these approaches have come to include many other elements such as communication skills building (Nielsen, 2017). Such integration results from cross‐pollination across the couple therapies (wise ideas become assimilated into other models) along with the powerful pragmatic issues which every couple therapist faces regardless of orientation such as how to manage spiraling angry interactions, engage the less invested partner in therapy, promote positive connection, or deal with comorbid individual emotional or physical health concerns.

Most approaches build from a biopsychosocial foundation that includes diverse aspects such as the influence of family history, cognition, emotion, and inner psychological processes. Thus, they tap into multiple levels of human experience (Lebow, 2014). For example, emotionally focused couple therapy (Greenman et al., 2019) addresses underlying primary and derivative emotions but also attachment. Enhanced cognitive behavioral therapy (Epstein & Baucom, 2002) addresses behavioral patterns but also relational schemas and emotions. Gottman method therapy (Gottman & Gottman, 2015, 2017) addresses the direct behavioral level of exchanges and a far deeper level of meaning. Integrative systemic therapy (Pinsof et al., 2018) addresses the many levels of human experience from behavioral exchange to inner experience.

Approaches certainly have differences in how much they emphasize each component (something we address later in this paper), but the overlap is considerable. Sometimes, authors explicitly speak of their approaches as integrative, while others do not; but regardless of whether they do so explicitly or not, integrative elements frequently permeate.

How should couple therapists think about and make use of these trends toward an expansion of both the specific phenomena to which contemporary approaches attend, as well as the broadening of various theoretical frameworks from which these phenomena are conceptualized? One approach that emerged during the 1970s was eclecticism—defined as the borrowing of specific techniques or constructs without allegiance (or even regard) for the theoretical framework in which those techniques or constructs were originally embedded (Lazarus, 1989). However, there are risks in eclecticism—most prominently the unsystematic or even contradictory use of specific interventions, as well as the possibility of dismantling interventions that rely on the synergistic effects of specific components implemented in combination for their effectiveness.

An alternative to eclecticism is pluralism—an approach that recognizes the validity and usefulness of multiple theoretical perspectives and draws on constructs and intervention strategies from across theoretical models by tailoring intervention strategies to a given case at any given moment based on their clinical relevance and potential utility. Pluralism differs from eclecticism in that interventions are always conceptualized from within a theoretical framework. Snyder (1999) advocated a pluralistic approach to couple therapy involving six levels progressing from a foundation of the collaborative alliance and managing initial crises, through strengthening the couple dyad and promoting relevant relationship skills, to addressing cognitive components and developmental sources of relationship distress. The therapeutic palette method of couple therapy presented by Fraenkel (2019) articulates a particularly elegant approach to pluralistic practice.

By the 1990s, the majority of therapists came to self‐identify as “integrative” rather than “eclectic” (even if their understanding of the difference might have been limited). Integration extends beyond pluralism via its blending of theoretical constructs or therapeutic techniques into one unified system or framework. Two threads of integration involve the identification of common factors and shared strategies, each of which we consider further here.

#### Common factors

A set of common factors lies at the base of couple therapy (Sprenkle et al., 2009). These include common factors shared with individual therapy such as the therapeutic alliance, the instillation of hope, and attending to feedback. There also is a second set of common factors unique to relational therapies that include maintaining a relational frame, an active therapy style, disrupting dysfunctional relationship patterns and supporting functional ones, and some effort to create a relational therapeutic alliance. Although not all models speak explicitly of common factors, most do attend to them. For example, it is rare to find an approach that does not include a discussion of creating a therapeutic alliance and attending to its complexities.

#### Shared strategies

Beyond common factors lies a wide array of strategies that either originated within one approach and migrated to other therapies or have emerged as important intervention pathways in different approaches (Lebow, 2014). For example, most approaches strive to promote some form of mutual empathy and understanding, some form of negotiation between partners, some engagement and focus on the strengths of the relationship, some affective reengagement of positive connection, some understanding of individual contributions to the conjoint problem, and some form of mindfulness or affect regulation to render conflict‐based interactions more constructive. Frequently shared strategies include tracking patterns, listening, witnessing, psychoeducation promoting mentalizing, promoting softening, and creating experiences that enhance attachment.

Notably, the naming of these shared strategies can often be a constraint in the recognition of shared ground. Terms such as cognitive restructuring, reframing, and restorying exemplify different jargon for similar interventions across approaches. Ironically, although apt and grounded in relational science, words that have come to be identified with specific theories such as attachment and differentiation often come to divide. Jargon readily invites a Tower of Babel in which similarities across approaches are not recognized and small differences in methods are accentuated over common ground (Miller et al., 1997). (Notable exceptions exist—for example, the use of the word “softening” in emotionally focused couple therapy has been enormously helpful in providing the perfect word for a broadly recognized intervention across diverse approaches.)

### Structure of sessions and other arrangements

Given the many different approaches to couple therapy and the varying problems and purposes for which it is employed, the extent of shared arrangements is quite remarkable. Couple therapy today is primarily done conjointly with a clear set of specified rules for separate communication with individual partners. Sessions are most commonly conducted for 1 hour per week, and most methods include some carryover of the process (e.g., homework) between sessions. Couple therapy may continue for only a few sessions or last years, but most models envision a process lasting between 3 and 12 months. It is striking that even though there have been innumerable methods developed that are aimed to be conducted over either briefer or longer timeframes (and even in the wake of randomized controlled trial protocols that often necessarily limit the number of sessions), and with shorter or lengthier sessions, the standard remains mostly the standard. Whether this is driven by custom, by cost considerations such as insurance reimbursement, or by some shared notion that this is most effective remains an open question.

### Couple therapies have evolved from their origins

Couple therapy models emerged out of various theoretical traditions, each anchored in its own time of development. However, it is in the nature of psychotherapies that, whereas theories and concepts often last over time, specific approaches do not. For example, behavioral marital therapy was initially a distinct, singular approach. That original treatment has been largely supplanted by the considerably expanded cognitive‐behavioral couple therapy (Epstein & Baucom, 2002) and integrative behavioral couple therapy (Christensen et al., 2020). Similarly, emotion‐focused therapy has been succeeded by emotionally focused couple therapy (Johnson, 2015) and emotion‐focused couple therapy (Goldman & Greenberg, 2015). In a like manner, early psychoanalytic therapies have been superseded by object relations couple therapy (Scharff & Scharff, 2005; Siegel, 2015) and mentalization‐based couple therapy (Bleiberg et al., 2023). And Bowen therapy (Bowen, 1972) and contextual therapy (Boszormenyi‐Nagy, 1987) have been largely supplanted by a broader more attachment‐oriented version of intergenerational therapy (Fishbane, 2019). Other early therapies, such as structural, experiential, and strategic couple therapy, have now declined in their prominence although they still have a cadre of devoted followers and their critical influence can be seen in various contemporary approaches. In tandem, the practice of some forms of couple therapy, such as narrative therapy (Freedman & Combs, 2015), has vastly expanded and evolved. And newer forms of couple therapy have emerged, such as socioculturally attuned couple therapy (McDowell et al., 2018) and acceptance and commitment couple therapy (Lawrence et al., 2023), as well as numerous specific therapies targeting specific issues or populations.

### A central role for culture and gender

Couple therapy began as “marital” therapy—that is, with a fixed set of ideas about who comprised the couple (a man and a woman), their legal status as a couple (married), and often with a stereotypic set of expectations having to do with roles and other aspects of the relationship. And from this perspective, marital therapy without much self‐reflection often spoke primarily to the experience of white, middle‐ and upper‐class Americans and Europeans. Feminist, queer, and multicultural perspectives, as well as the dissemination of couple therapy around the world, have very much changed this perspective (Addison & Coolhart, 2015; Kelly et al., 2019). Couple therapy is now a vehicle for helping with intimate relationships across gender, sexual preference, class, culture, race, ethnicity, and other facets of social location.

Understanding couples in the context of culture, race, ethnicity, gender, sexual orientation, and other aspects of social location that afford persons greater or less privilege (and greater or lesser experiences of marginalization and oppression) has become an essential aspect of couple therapy. Further, couple therapies are most helpful when adapted to specific kinds of couples—for example, adaptations for LGBTQ couples (Coolhart, 2023; Green & Mitchell, 2015) and stepfamily couples (Papernow, 2018a), or description of the special considerations in therapy with Black American couples (Kelly et al., 2019) or Latinx couples (Falicov, 2014). These insights and practices do not require clinicians to relinquish their favored theoretical approach to couple therapy but do present crucial additional considerations in the context of working with couples in a sensitive and effective manner.

## COMMON ELEMENTS OF COUPLE THERAPY

### Assessment

Assessing multiple domains (e.g., emotions, cognitions, and behaviors) across multiple system levels (e.g., individual partners, their relationships, and broader family and cultural contexts) is essential for selecting, tailoring, and sequencing couple therapy interventions in a planful and effective manner. Whether implicitly or explicitly, the different approaches universally recognize the importance of attending to individual differences in conducting relevant interventions. Similarly, nearly all speak to the importance of monitoring both the process and progress of therapy in evaluating the impact of specific interventions, and revising the clinical formulation (whether explicit or implicit) and plan of therapy accordingly.

That said, both theoretical models and specific applications of couple therapy vary in their philosophical stance toward normative versus idiographic approaches, their advocacy of specific content or methods, and their views on whether formal assessment necessarily precedes intervention or, instead, evolves organically throughout therapy. Some approaches advocate meticulous assessment and the generation of an explicit case formulation and treatment plan (Christensen et al., 2020), whereas some others do not. Some approaches such as narrative therapy explicitly eschew specific assessment methods (Freedman & Combs, 2015). And among those approaches that purposely incorporate methods of assessment, there may be a formal stage of assessment (e.g., a four‐session protocol combining individual and conjoint meetings; Chambers, 2012) or not; similarly, the various approaches or specific applications may prescribe standardized questionnaires or a set of observational tasks (Gottman, 1999; Gottman & Gottman, 2015) or not.

Related to assessment is the specification of specific inclusionary or (more usually) exclusionary criteria for couple therapy. Most models of couple therapy consider moderate to severe partner aggression, active alcohol or other substance abuse, continuing infidelity, or psychotic symptoms as contraindications for conjoint couple therapy. Yet, paradoxically, there are specific couple‐based treatments for these issues such as treatments for couples that include a person with a substance use disorder (McCrady et al., 2016) or infidelity (Baucom et al., 2009; Scheinkman & Werneck, 2010). A careful assessment facilitates informed decisions as to whether any of these or similar problems can be addressed within the more general theoretical models of couple therapy or require the more specialized intervention protocols, or whether any couple therapy is likely to be unhelpful in a particular case.

### A myriad of strategies of intervention and techniques

One marvels at the rich and distinct body of intervention methods that have been developed. Clearly, some of the most creative and astute clinicians have developed this wonderful array of methods. The various models for helping couples bubble over with a panoply of active ingredients couple therapists can incorporate into treatment. That said, effective therapists often come up with very similar ways of working in couple therapy across whatever divides exist among theories. Clearly, there also has been cross‐pollination.

### The systemic view: Sequences and vulnerability cycles

One important shared emphasis of almost all couple therapies lies in tracing the interpersonal sequences that unfold in the process of developing relational difficulties. This speaks to the influence of shared systemic understandings. Although certain processes may lie within individuals, the inevitable mutual influences between partners define the crucial understanding that is foundational to treating couples. It is in the nature of intimate relationships that the thoughts, feelings, and behaviors of partners inevitably affect one another and their relationship in an ongoing, recursive manner.

These cycles are named in a variety of ways across approaches, and what is seen as the specific internal component of the greatest moment in these cycles varies from approach to approach. Thus, Scheinkman and Fishbane (2004) speak of the vulnerability cycle, whereas Johnson and colleagues refer in their discussion of emotionally focused therapy to mutual attachment injuries (Johnson, 2015). In describing integrative systemic therapy, Pinsof et al. (2018) refer to sequences. Regardless of how these processes are named, the core sequence being referenced here involves a multilevel interpersonal process in which distressed partners turn away from one another or aggressively vie for control as opposed to engaging compassionately. The various general models of couple therapy articulate how these processes, like rust corroding the foundation of bridges, can erode the positive connection between partners. These models of couple therapy describe both how couples can develop and maintain a vital loving connection as well as the processes by which such connections diminish. Similarly, couple therapies targeted at specific problems and issues (e.g., post‐traumatic stress disorder or sexuality) emphasize how those issues come to be interwoven in the broader fabric of individual and relational functioning.

### Pragmatic focus on relationship satisfaction

Another clear point of overlap lies in a dual focus on reducing couple distress and promoting relationship satisfaction. Almost all couple therapies emphasize specific interventions targeting these two complementary outcomes. That said, models vary in their relative emphasis on one versus the other. By definition, couple‐based applications for specific relationship issues (e.g., partner aggression or infidelity) or individual problems (e.g., depression or anxiety disorders, alcohol problems, and acute medical issues) target reduction in these difficulties, with improvement in relationship satisfaction often being viewed as one of the mediating pathways. Historically, many couple therapies have focused more on reducing conflict than on promoting intimacy—although more recently such positive aspects of relationships as encouraging emotional connection and shared meaning have moved into greater focus. Theories of couple functioning and related models of intervention play a pivotal role through their differential emphasis on specific aspects of relationships such as attachment, mentalization, mutual acceptance, problem‐solving and communication, narratives, and gender or sociocultural consciousness.

### Ethical considerations

Couple therapists across orientations recognize a shared set of ethical considerations. Although couple therapies may disagree about what is the optimal ethical decision in a specific circumstance (e.g., whether to hold certain secrets—most especially about past behavior), there is almost total agreement on where the ethical issues lie and how to think about those issues. Thus, discussions about ethics in couple therapy speak to almost all couple therapies regardless of the specific application or underlying theoretical model (Barnett & Jacobson, 2019; Gottlieb et al., 2008; Margolin et al., 2023). Couple therapists struggle with the same complex set of dilemmas and questions, and most often come up with similar answers about such issues as confidentiality about private communication with one partner during couple therapy; about identifying who the client is in therapy, and how to respond to one partner's desire to leave the relationship; or about how to deal with the risk of intimate partner violence. Sometimes, there are differences about what is to be done in a specific circumstance; however, it is rare for an idea about these issues to be presented without recognizing that others may hold different positions and an awareness of the complexities involved in holding particular positions. Nonetheless, we must remember that in the practice of such a complex endeavor as couple therapy, there always will be those who are exceptions in their beliefs about some debatable standards of good practice.

### Relation to individual and family therapy

Even as couple therapy has differentiated itself from individual and family therapy, it also has found a place to incorporate these modalities. In relation to individual therapy, most of the methods co‐exist and often actively look to be enhanced through collateral work with an individual partner. Although in some models that “individual” work may be done within the couple format, many suggest a complementary role for concurrent individual therapy with a different therapist.

Ironically, given its systemic roots, concurrent family therapy is less frequently spoken of in expositions about couple therapy than is individual therapy. Some approaches do retain the fluidity between couple and family therapy (at least in the unit of focus in therapy). Intergenerational approaches include a considerable focus on the family of origin and some still bring the family of origin into couple therapy sessions (Fishbane, 2019). Family systems considerations focused on children also become a center of attention in considering couple distress in the special circumstance of working with couples in which one partner leans toward ending the relationship while the other wants to continue with it before making a decision to enter couple therapy, where the impact on children typically arises as an important factor (Doherty & Harris, 2017). Additionally, Wymbs et al. (2023) speak to the role of working with couples as part of a multiformat approach with families of youth with attention deficit hyperactivity disorder or disruptive behavior disorders. Similarly, in their discussion of therapy with couples with medical issues, Rolland (2019) and Ruddy and McDaniel (2015) describe how that approach derives from broader medical family therapy. Notably, some of the most popular forms of couple therapy such as emotionally focused couple therapy have recently spawned related forms of individual and family therapy (Furrow et al., 2019).

### Stages of couple therapy

Although there are exceptions, most couple therapies envision beginning therapy with a stage of assessment and building of the therapeutic alliance, followed by a stage of promoting change (e.g., reducing couple distress and fostering positive connection), and then a concluding stage of termination and maintenance of gains. In the initial stage, many approaches include an explicit sharing or co‐creation of the clinical formulation and tentative treatment plan, reflecting emerging emphases in the field on collaboration and transparency in all phases of couple therapy.

## FACETS OF DIFFERENCES ACROSS APPROACHES

Despite the underlying pragmatism and integration evident in many contemporary couple therapies, theories do matter. In his seminal 1978 analysis, Alan Gurman spelled out the essential tenets of what then were the major schools of couple therapy: behavioral, psychoanalytic, and systemic approaches (Gurman, 1978). In this classic deconstruction of couple therapies, Gurman differentiated couple therapies along four dimensions: (1) the role of the past and of the unconscious; (2) the nature and meaning of presenting problems and the role of assessment; (3) the relative importance of mediating versus ultimate treatment goals; and (4) the nature of the therapist's roles and functions. Fraenkel (2009), following a similar analysis, highlighted that approaches differ in (1) time frame (present, past, or future), (2) change entry point (thoughts, emotion, or behavior), and (3) degree of directiveness. It is striking (although perhaps not surprising) that now, decades later, these key facets of differences still apply today.

Earlier, we noted multiple sources of commonality across couple therapies—including shared systemic understandings, integration of specific techniques across approaches (even if reconceptualized within an alternative theoretical framework), the broadening of therapeutic focus (i.e., the near‐universal consideration of thoughts, feelings, and behaviors), and common arrangements (e.g., the emphasis on conjoint sessions). That said, while sharing considerable foundational elements, couple therapies in the 21st century can be differentiated along multiple dimensions—including (but extending beyond) those cited in previous analyses—both in terms of unique components as well as their relative emphasis on various shared components. Below, we summarize some of the most important, differentiating facets of various couple therapies.

### The defining elements of a successful relationship

What are the most essential features that define a successful couple relationship? What are the typical individual elements, relationship patterns, or broader systemic characteristics that differentiate healthy or well‐functioning couples from those challenged by distress or dysfunction? Relatedly, what implicit or explicit theory of love and connection underlies a particular therapeutic model? For some, the answer lies in growing the couple friendship; for others, in attachment; for others, in how partners think and feel about their relationship; for others, the broader historical or cultural context; for some, sexuality; and, for still others, deep intrapsychic needs and capacities to connect. For some, peak experiences (and intensity of connection) are stressed (Perel, 2006); for others, steadiness and order. Although it is now typical for various models to speak to multiple levels of experience, the therapeutic approaches to couple therapy tend to emphasize one predominant lens in their theory of love, connection, and health.

### Whom to include in the couple therapy

As noted earlier, contemporary approaches typically operationalize couple therapy as uniquely involving conjoint sessions with two relationship partners. That said, there are important exceptions. For example, many theoretical models and specific applications advocate for the inclusion of individual interviews during the initial assessment—particularly as opportunities for partners to discuss topics they may not yet feel comfortable discussing in the presence of their partner (e.g., infidelity, intimate partner violence, or considerations of divorce). Specific policies for handling confidential communication in such individual meetings may also vary across approaches (Scheinkman, 2019; Scheinkman & Werneck, 2010). Some suggest infusing individual sessions during the couple therapy as a means for disrupting unremitting, escalating negative exchanges until better self‐regulation can be achieved with the individual partners and then incorporating that individual work into resumed conjoint sessions. Some models have more flexible boundaries about whom to include, based on whomever the therapist or partners regard as potentially helpful in the process of improving the relationship. For example, members of the extended family may be included occasionally in integrative systemic therapy (Pinsof et al., 2018) and intergenerational couple therapy (Fishbane, 2019). Papernow (2018a) notes that ex‐spouses are a permanent part of the family; hence, couple therapists may need to incorporate time‐limited intervention with ex‐spouses to promote more collaborative coparenting across households. In approaches to polyamorous relationships there may be little or no hierarchy, and all relationships may be treated as equally important (Coolhart, 2023); within that context, discussions of interpartner conflict, attachment, security, jealousy, or relationship roles and boundaries easily require reconfiguration of couple therapy from a dyadic to a broader multipartner context.

Separate from issues of “whom to include” is the setting for the couple work. At the pragmatic level, where to conduct the therapy may be influenced by medical issues, mobility, systemic constraints (e.g., access to childcare or transportation), and a host of related concerns. Telehealth has recently emerged as a primary mode for the delivery of couple therapy (see below) (Fraenkel & Cho, 2020; Hardy et al., 2021). Telehealth may reduce but not eliminate constraints in access, depending on access to, and proficiency with, relevant technology. Approaches to couple therapy also vary in how much they consider the couple “work” to extend outside of sessions to between‐session (e.g., at‐home) prescribed exercises or enactments and the use of such materials as worksheets or ancillary texts.

### The role of the therapist

The role of the couple therapist represents an aspect of therapy about which there is considerable debate. Certainly, all acknowledge the therapist as a vital part of a system with the couple, and all accentuate the importance of alliance and collaboration. That said, the various models differ in how they regard the therapist's position in relation to both partners and the roles they ideally fulfill.

#### Influences on the therapeutic process

Although the various approaches to couple therapy universally recognize the importance of the therapeutic alliance as a common factor (Sprenkle et al., 2009), they differ considerably in how they envision the therapist influencing (and being influenced by) the therapeutic process. There was a time when couple therapy largely consisted of therapists assuming the role of an expert in teaching partners about how to pursue a more functional relationship. Although this instructional role of the therapist remains a thread in the work of several approaches (such as cognitive‐behavioral couple therapy and Gottman method therapy) as well as in the applications of couple therapy to specific relational issues or individual problems, more broadly the field has moved from hierarchical therapist–couple relationships toward a much more collaborative stance. For example, some couple therapy models such as solution‐focused, narrative, and the therapeutic palette emphasize the therapist's and couple's collaborative co‐construction of the treatment goals and strategies, during which the therapist participates as a “fellow traveler” who facilitates the partners' realization of their own unique goals and pathways toward attaining these (Freedman & Combs, 2015). Most approaches locate themselves somewhere midway along the continuum between expert guide and fellow sojourner.

#### Attention to self of the therapist

Couple therapies also vary in how much they attend to the “self of the therapist” as an integral component of the therapy process. From this perspective, therapists need to pursue mindfulness of their own thoughts and emotions, memories, values, and implicit assumptions or biases to draw on both their past and present experiences in relating and intervening with couples (Aponte & Kissil, 2016). Some models emphasize such self‐awareness as an essential core component of effective therapy—for example, socioculturally attuned couple therapy (McDowell et al., 2018) and object relations couple therapy (Scharff & Scharff, 2005; Siegel, 2015), as well as couple therapies tailored to populations where issues of identity are often central such as LBGTQ couples (Coolhart, 2023), and couples from specific ethnic or racial cultural contexts (Falicov, 2014; Kelly et al., 2019).

Notably, approaches that once most centrally emphasized the self of the therapist and therapist self‐disclosure (e.g., Whitaker's symbolic‐experiential therapy; Whitaker, 1958; Whitaker & Keith, 1981) now play a less prominent role in couple therapy. It is also notable that whereas many early models explicitly called on therapists in training to participate themselves in couple therapy, we have been unable to locate recent writing specifically about couple therapy that does so, despite its obvious potential value.

Some approaches encourage therapist self‐disclosure, whereas many others do not. Most models leave open the possibility without being explicit about guidelines for self‐disclosure. Yet, transcending these differences, most approaches encourage therapists to recognize and draw upon their own subjective experiences during the therapy process (e.g., feelings of empathy, irritation, or boredom) as important information regarding the content and process of interactions with the couple or between partners themselves.

### Levels and focus of interventions

By definition, couple therapies focus on the couple dyad and, for the most part, on the aggregate subjective balance of couple distress versus well‐being. However, within that general framework, approaches vary considerably in their consideration of multiple system levels including individual partner characteristics, aspects of the extended family, and the broader socioecological context. Approaches also vary in their relative emphasis on emotions, cognitions, and behaviors—and the explanatory or conceptual lens through which each of these are understood. And there are marked differences in the order of intervention even when there is a shared base of strategies. For example, integrative systemic therapy suggests first dealing with action‐oriented aspects of the relationship whereas integrative behavior couple therapy (Christensen et al., 2020) first accentuates acceptance and Nielsen's integrative approach (Nielsen, 2017) prioritizes understanding underlying issues in the relationship.

#### Levels of intervention

Contemporary approaches to couple therapy all share a systemic perspective, but with varying points of emphasis. For some, there is a greater focus on individual processes. For example, in object relations therapy (Scharff & Scharff, 2005; Siegel, 2015) and intergenerational approaches to couple therapy (Fishbane, 2019) the enduring and predisposing vulnerabilities of the individual partners, rooted in their respective family and prior relationship histories, comprise the foundational substrate from which interactive vulnerabilities, self‐ and partner perceptions, and exaggerated response dispositions evolve. By contrast, other therapies focus less on the individual partners, and more on sequences of interaction (Hoyt, 2015). Still, others place greater emphasis on contextual factors as contributing or perpetuating influences on couple distress or dysfunction. From this perspective, such influences as systemic poverty, racism, or heterosexist and cisgender bias not only moderate the development or treatment of couple distress—they directly contribute to it (Hardy & Bobes, 2017; Knudson‐Martin & Kim, 2023) and, hence, comprise a central focus of treatment.

Moreover, the various approaches may target individual problems, relational problems, broader systemic influences, or any combination of these—either in their underlying theoretical formulation or in their specific application (as in the application of cognitive–behavioral couple therapy to individual disorders).

#### Focus of intervention

Similarly, contemporary couple therapies vary in their relative focus on specific areas of content, regardless of the system level of intervention. Most all recognize the interactions among thoughts, feelings, and behaviors, but their emphases on one or another of these domains differ considerably. Even the labeling of the approaches reflects these differences—for example, the naming of cognitive‐behavioral versus emotionally focused couple therapy. Further, there is an argument even across approaches that target multiple dimensions of experience about how the optimal sequence for addressing these should proceed. For example, some suggest behavior should be addressed first (e.g., integrative systemic therapy) whereas others initially emphasize such processes as attachment (e.g., as in emotionally focused couple therapy) or acceptance (e.g., as in integrative‐behavioral couple therapy). Moreover, partners may be encouraged to attend primarily to the subjective experiences of the other (e.g., to promote empathic awareness and joining) or, instead, to pursue mindfulness of their own thoughts and feelings as these influence relational exchanges (e.g., as in acceptance and commitment couple therapy).

Also influencing the content of interventions are approaches' differential attention to levels of awareness related to subjective thoughts and feelings. For example, partners' expectations of themselves and each other may reside well within conscious awareness, may lie outside immediate awareness but prove accessible with modest guidance from a cognitive framework, or may rely upon techniques more typical of various psychodynamic approaches for uncovering latent internal processes and explicating their influence in the current relationship. Sager's (1976) work on such “hidden forces” in couple relationships, and their impact on both implicit and explicit contracts (and their degrees of congruence or discordance), offered an influential explication of levels of consciousness as related to different approaches to intervention and provides a useful lens to inform such considerations.

The various approaches to couple therapy also differ considerably in their relative emphases on overt change (e.g., cognitive‐behavioral and solution‐focused couple therapy) versus acceptance (e.g., integrative behavioral couple therapy). Notably, even among those therapies that emphasize acceptance, approaches vary in how they conceptualize and promote this outcome. For example, in integrative behavioral couple therapy, acceptance is pursued through specific interventions promoting empathic joining (emotional change) and unified detachment (cognitive change) as an alternative (or precursor) to interventions targeting behavioral change. In acceptance and commitment therapy (Lawrence et al., 2023), partners are encouraged to experience uncomfortable internal experiences and to tolerate their presence rather than trying to control them, so that they can allocate their time, energy, and attention in more fulfilling ways. In the various psychodynamic and multigenerational approaches, partners' acceptance evolves from changes in understandings of their own and each other's developmental histories and associated vulnerabilities—that is, through partners' more compassionate interpretations or meanings (and hence, related feelings) connected to specific behaviors or interaction sequences.

#### Presumed mechanisms of change

Closely related to levels and focus of interventions are the various approaches' underlying theoretical tenets regarding mechanisms of change. Separate from their shared emphasis on the therapeutic alliance, most approaches first prioritize attending to disabling individual or relationship crises. Beyond such shared initial “stabilization” interventions, however, the various approaches' theoretical precepts guide the selection, sequencing, and even pacing of specific interventions. Some models, for example, prioritize behavior change (or problem solutions) as the mediating pathway for promoting partners' positive thoughts and feelings for one another. Others prioritize interventions aimed at altering partners' thoughts toward one another—including the interpretations or meaning they give to relational events (whether explicit or implicit) as the mediating pathway for reducing negative affect derived from the subjective meaning and, by reducing subjective negativity, thereby fostering more positive exchanges. And still other approaches prioritize interventions aimed at promoting emotional connection (e.g., via vulnerable emotional expression and empathic responding) or acceptance (e.g., tolerance of inevitable differences). From any of the pluralistic or integrative approaches, the therapist could select specific interventions from across theoretical models, based on their presumed mechanism of change and in congruence with the case formulation.

### The temporal framework of interventions

How important is the exploration of partners' individual and shared histories? Some approaches, such as intergenerational ones are fully anchored in the past and may begin with genograms as both an assessment and intervention method. Others, such as solution‐focused therapy (Hoyt, 2015) are almost exclusively present focused. Most contemporary couple therapies incorporate attention to both distal (historical) and more proximal (recent or current) influences, although often to different degrees or in different sequences. For example, in Snyder's (1999) pluralistic approach, developmental influences are pursued only after more structural or cognitive‐behavioral interventions fail to achieve desired outcomes. Moreover, in various integrative approaches or specific theoretical models that assimilate particular techniques from alternative approaches, the labeling of techniques or their interpretation through a particular theoretical lens may obscure similarities in their application (e.g., identifying projective identifications in object relations therapy, attachment injuries in emotionally focused therapy, or acquired perceptual and behavioral response dispositions in cognitive‐behavioral couple therapy).

### Manualized versus improvisational approaches

Contemporary couple therapies vary in their level of structure. Some therapies are highly improvisational; Fraenkel (2019), for example, even names improvisation as a core aspect of the therapy. Others are much more prescriptive regarding the sequence and general content of interventions—e.g., couple therapy for partner aggression (Epstein et al., 2015) or infidelity (Baucom et al., 2009). Some approaches—e.g., Gottman method therapy (Gottman & Gottman, 2015, 2017) and Papernow's therapy for stepfamily couples (Papernow, 2018b) propose specific goals of intervention and methods of accomplishing those goals, although the sequence and number of sessions devoted to each goal may be tailored to aspects of the individual partners and their relationship. Applications of couple therapy to individual problems such as posttraumatic stress disorder or alcohol abuse, similar to their cognitive‐behavioral counterparts in individual therapy, tend to be more highly structured or manualized—often with a specific sequence and prescribed “curriculum” detailing specific sessions.

### Length of therapy and intermediate versus ultimate goals

Couple therapy can be open‐ended or time‐limited. Solution‐focused couple therapy (Hoyt, 2015) anchors this continuum through its explicit focus on brief interventions targeting circumscribed problems. Other couple therapies of all varieties may segue into ongoing meetings over many years, potentially reflecting a transition from initial interventions promoting specific relationship skills to a subsequent emphasis on partners' individual growth within a conjoint framework. Most contemporary couple therapies terminate after sufficient progress toward initial goals has been achieved. Longer durations can be anticipated, regardless of approach, with couples for whom individual, relational, or broader systemic dysfunctions are more severe, more complex or pervasive across multiple domains, or more entrenched across time.

Gurman's (1978) distinction between mediating versus ultimate treatment goals also provides a useful heuristic for viewing shorter‐ versus longer‐term approaches. For example, when situational stressors compromise partners' functioning and couple well‐being, initial goals may involve resolving those stressors to achieve a direct (and potentially sufficient) effect on reducing couple distress (Bodenmann & Randall, 2020). However, if in the course of that work the therapist determined that traumatic individual developmental experiences mediated the impact of current stressors on individual and relational functioning, then stress‐reduction might shift to being an intermediate goal and the “ultimate” goal might be reconceptualized as emotional or cognitive reprocessing of traumatic experiences to reduce or resolve their contribution to recurrent patterns of vulnerability or exaggerated reactivity. In the final analysis, the formulation of treatment goals and related decisions about termination inevitably reflect an evolving interaction between the therapeutic approach and couples' own values, aspirations, and resources.

## EMERGING ELEMENTS

There also are emerging an exciting array of novel elements in contemporary couple therapies.

### Technology

The Covid‐19 pandemic potentiated a trend already developing in couple therapy toward telehealth and using electronic media as extensions of therapy. Much of couple therapy delivered during the pandemic shifted to videoconferencing and it appears that videoconferencing will remain a major format for couple therapy. Therapists needed to augment and adapt their methods to a context during which in‐person meetings were not possible. Fairly quickly, several useful sets of guidelines for relational teletherapy were offered (Burgoyne & Cohn, 2020; Drieves, 2021; Hardy et al., 2021; Hertlein et al., 2021). Couple therapists mostly report that virtual therapy appears to work as well as in‐person therapy (de Boer et al., 2021).^1^Additionally, video‐conference couple therapy sometimes may be the sole viable alternative to in‐person sessions (e.g., when partners are geographically separated by work, deployment, or other factors). Videoconferencing solves one of the major constraints of couple therapy that historically had caused so many who could benefit from couple therapy not to seek it—namely, individual control over the time and place of meeting. For many persons, meeting virtually from their homes or from work is easier, and therapists can often be more flexible with the scheduling of sessions in this format. It can be relatively easy to assemble a couple in virtual space, and often much harder to do so in person. (It must also be added that for some, such as many older and economically disadvantaged potential clients, videconferencing makes for an additional constraint in accessibility.)

Many recent writings about couple therapy refer to these now ubiquitous methods of videoconferencing. There has yet to be much written about special issues that arise in couple video therapy such as special methods for working with conflict at a distance, guidelines for working with intimate partner violence, and privacy issues. As to the outcomes of video‐conference couple therapy compared to in‐person couple therapy, we must await the data, not only for the global question of impact but also for whether there are differences in impact across types of couples (e.g., by problem area or demographics), as well as for process data such as the quality of the therapeutic alliance across these formats.

Beyond using videoconferencing services for couple therapy, there is considerable growing excitement regarding the application of web‐based resources as adjuncts to treatment (Hatch et al., 2021; Roddy et al., 2016, 2021) or in relationship education (Bradbury & Bodenmann, 2020; Markman et al., 2022; Rohrbaugh, 2021; Spencer & Anderson, 2021). Models on the technological cutting edge such as Gottman method therapy now regularly augment couple therapy with online psychoeducational materials, reminders to engage in prescribed behaviors, and even physiological measures of partners' autonomic arousal.

### Couple therapy and social media

Couple therapy is increasingly an evidence‐based practice. Yet, in tandem, couple therapy now is frequently identified by lay consumers not by its evidence‐based variations but through its dissemination through popular media. The extent to which those representations of couple therapy are grounded in the state‐of‐the‐art practice of couple therapy varies. For example, Perel (2006) builds from well‐known traditions from psychoanalytic couple therapy and systemic practice. Real (2008) similarly builds on the traditions of feminist couple therapy and treatment of relational trauma. And Gottman and Gottman (2015) and Johnson (2015), developers of major forms of couple therapy, have crossed over into providing highly accessible aspects of couple therapy in podcasts and other new media. Similarly, Solomon et al. (2021) has adapted and popularized a version of integrative systemic therapy in her approach to young people in relationships. Still, one cannot help but note that there are innumerable examples of well‐known persons and internet personalities suddenly turning into relationship coaches offering advice, based on their personal notion of how to live a relational life (not surprisingly, most of these lean toward the dramatic). Similarly, some of the best‐selling guides for couples (e.g., Gray's 1992 “Men Are from Mars, Women Are from Venus” and Chapman's 1992 “The Five Love Languages”) are inconsistent with research from relational science. It is a time of much attention to couple therapy, and a time in which having informed consumers is essential to helping potential clients separate what is grounded and what is performance.

### Specific treatments for specific problems and populations

Couple therapy has traditionally been mostly envisioned as a process targeted at improving relationship satisfaction or, at least, as deciphering the viability of committed relationships. However, over the last 20 years, couple therapies have been developed and widely disseminated focusing on problems traditionally viewed as residing within individuals. Baucom et al. (2014) provide a useful distinction between partner‐assisted and disorder‐focused interventions targeted at individual problems. In partner‐assisted interventions, the partner is enlisted to help in the process of reinforcing and supporting the active treatment of the individual problem. In contrast, in disorder‐specific treatment, the treatment itself is couple therapy tailored to the particular kinds of couple dynamics likely to occur in the context of the partner's individual problem.

Today, given the predominance of cognitive behavioral therapies for the treatment of individual disorders, couple treatments of individual problems are also mostly cognitive‐behavioral in their approach. However, other models, such as emotionally focused couple therapy, have begun to speak to such uses of couple therapy across several specific disorders (Slootmaeckers & Migerode, 2020) and one could anticipate that such applications of other theoretical models of couple therapy to treat individual emotional or physical‐health problems will continue to proliferate.

Couples often present for therapy to receive assistance with issues around parenting of their children or adolescents. Traditional parenting programs, while promoting positivity in parent–child interactions, give only limited attention to the relationship between parents. Many family therapy models for parents and adolescents with various disorders (e.g., conduct disorder or substance misuse) also under‐attend to the couple relationship itself and its recursive influences upon and from the adolescent's behaviors. It is inevitable that parents will experience occasions of disagreement or other challenges when rearing children together. Couple challenges associated with children's behaviors become more frequent, severe, and difficult to resolve when offspring have their own individual problems—whether these take the form of internalizing, externalizing, or neurodevelopmental disorders. Expositions of couple therapy with parents of youth with emotional or behavioral disorders have been notably rare, and there is a need for a general framework for tailoring interventions to couples struggling with these common concerns.

### Reaching out to a wider range of couples

As culture and gender have become more central considerations in couple therapy, approaches explicitly addressing issues of diversity have also emerged and gained broader traction. Exemplars include the discussions of therapy with LGBTQ couples (Coolhart, 2023; Green & Mitchell, 2015), interventions involving sexuality (Hall & Watter, 2023), and therapy targeted to couples from specific ethnic groups (Boyd‐Franklin et al., 2008; Chambers, 2019; Falicov, 2014; Kelly et al., 2020). One cannot underestimate the sea change that has been involved.^2^ Generalizations about couples and about the most helpful interventions with them are now enhanced with a far greater appreciation of differences among couples and how those can best be attended to.

Old formulations of relationships or guidelines for therapy must now be viewed through new lenses. The evolution in the breadth of couples embraced by the field of couple therapy has been enormous. For example, today, nearly all theoretical approaches to couple therapy explicitly address issues of applicability to LGBTQ couples and most have begun to stretch to include the emerging broader world of sexuality in couples. This broadening of the vision of who is involved in couple therapy has also unearthed culture‐bound assumptions and led to adaptations and advances in the core models of couple therapy in both their development and delivery.

### The Interface with relationship education

Relationship education has a long and distinguished history as it developed in parallel with couple therapy (Bradbury & Bodenmann, 2020; Markman et al., 2022). Relationship education and enrichment programs of late have become ubiquitous. This has promoted lively conversations about which couples (or individual partners) are most appropriate for which activity, about the fuzzy boundaries between education and treatment, and how to manage or optimize the interface between them. Whereas at one time it was clear that couple therapy was targeted at distressed couples and relationship education aimed at preparation and enrichment of better functioning relationships, this boundary has become much more fluid (Bradford et al., 2015). Further, several models of couple therapy —e.g., integrative behavioral couple therapy (Roddy et al., 2017) and emotionally focused couple therapy (Conradi et al., 2018)— describe adaptations of those models intended for either in‐person, videoconference, or self‐directed online psychoeducational relationship education programs. And there is a growing movement toward relationship education involving individuals not presently in relationships so that they might develop healthier relationships (Carlson et al., 2023).

### The growing emphasis on acceptance

Acceptance has moved into a much more prominent place in several methods of couple therapy, including integrative‐behavioral couple therapy, Gottman method therapy, acceptance and commitment couple therapy, and mentalization‐based couple therapy. At one time, change was the focus of every couple therapy; now, many seek primarily to promote mutual acceptance while also facilitating a framework for change.

### Collaborative therapists

Overall, the field has moved from implicit views of a somewhat hierarchical therapist–couple relationship toward a much more collaborative stance. A collaborative stance goes well beyond elements of promoting a therapeutic alliance initially identified in client‐centered individual therapy (i.e., genuineness, warmth, and noncontingent positive regard). Rather, collaboration extends to co‐constructing therapeutic goals that incorporate partners' own views of individual and relationship health, their values rooted in their unique developmental histories and broader cultural contexts, and their own priorities regarding the balancing of individual with relationship interests in determining how to select and sequence treatment objectives and methods.

### Addressing sexuality

Sexuality is clearly a central aspect of relational life, both in itself and in its association with attachment. Hence, it is somewhat bewildering why, in most models of couple therapy, it is so tangentially addressed. Notably, this core component of relationships is principally addressed in specific discussions of sexuality (Hall & Watter, 2023; McCarthy & McCarthy, 2012; Perel, 2006) and often about LGBTQ couples (Coolhart, 2023). Despite the limited attention to sexuality in many treatment models, there has been a revolution in the consideration of sexuality when working with couples. Couple therapists need to challenge their own implicit attitudes or assumptions, and expand their knowledge base and skill sets, when addressing sexuality in working with sexual‐ and gender‐minoritized couples. Similarly, therapists need to become familiar with and comfortable in discussing aspects of sexuality that may vary in specific populations—such as older adults, couples confronting specific medical problems, or couples who engage in less frequently encountered forms of sexuality. Couple therapy around issues of sexuality has evolved beyond addressing specific sexual dysfunctions and, instead, now embraces broader goals of promoting greater sexual awareness, improving sexual responsiveness, and enhancing sexual intimacy and enjoyment that might benefit any couple.

### Attending to the life cycle

Both the challenges and benefits of being a couple vary across the life cycle. Most models of couple therapy have implicitly centered on mid‐life couples, and the specific issues and intervention strategies they emphasize do not always generalize to younger couples early in their individual and relational development, nor to older couples for whom individual and relational challenges and resources often change. The good news here is that many models have now evolved to incorporate couple development over time as a part of their vision. Beyond this, there is an emerging increased focus on specific stages of development and the typical issues in couples related to those life stages (see, e.g., Solomon et al., 2021 on emerging adults; and Knight, 2023 on older adults). These include attention to special issues in older couples, the unique issues and challenges that confront stepfamily couples, and younger couples—particularly around decisions to formalize a committed relationship or transition to parenthood. Specific couple interventions have been developed for working with couples in specific stages of the life cycle (Gottman et al., 2010). From a broader perspective, the question of how to keep relationships vital and connected over a lifetime underlies most couple therapy.

### Divorce

Whither divorce in couple therapy? Long regarded as a disastrous negative outcome, divorce is now re‐envisioned as a potential positive pathway for couples, yet one fraught with challenges. New versions of intervention have recently been developed to help couples who face the possibility of divorce. For example, Doherty and Harris (2017) offer discernment counseling targeted to those not yet ready for couple therapy who are ambivalent or have mixed agendas about whether they want to divorce, to help the partners decide on whether working on their relationship further in couple therapy is indicated. How to work with those considering divorce, with the therapist finding a balanced position toward couples remaining together or parting, has become an essential aspect of couple therapy. So too has helping those who decide to divorce to pursue the best outcomes for themselves and for the children who may be impacted (Lebow, 2019). Couples often envision couple therapy ending if they decide to divorce, but “divorce therapy” is paradoxically an essential part of the repertoire of the skilled couple therapist.

Closely related are therapies focused on what Fraenkel (2019) calls “last chance” couples. These couples are already on the verge of divorce and, if therapy is to reinvent the relationship, a more radical process may be needed than in typical couple therapy.

## ADDITIONAL CHALLENGES

Contemporary couple therapies face numerous challenges—some enduring since the inception of the field (e.g., attention to individual differences and issues of diversity; balancing interventions to address intrapersonal, dyadic, and broader systemic sources of distress)—and others more recent (e.g., integrating technology; securing recognition across private and public healthcare systems). Some challenges are either explicit or implicit in earlier parts of this paper (e.g., decisions regarding whom to include in the couple therapy; the balancing of acceptance versus change; or specific ethical dilemmas). Beyond these, two additional challenges warrant consideration.

### Maintenance of gains

One crucial challenge for couple therapy is the maintenance of therapeutic gains. Research has shown couple therapy to be highly effective in improving relationship satisfaction in most couples in the short term (Bradbury & Bodenmann, 2020; Roddy et al., 2020), but vulnerable to problems returning over the long term (i.e., at 2 years or longer after termination). From the few controlled clinical trials of couple therapy and one uncontrolled evaluation examining couple outcomes 4–5 years after posttreatment, the evidence shows deterioration or divorce occurring for roughly 35%–50% of couples (Snyder & Balderrama‐Durbin, 2020). Exceptions to this general finding, such as Snyder et al.'s (1991) controlled trial of insight‐oriented therapy yielding a deterioration/divorce rate of 20% at 4 years posttreatment, have not been replicated.

Moreover, couple relationships evolve and different stages of the life cycle begat different problems. Thus, it would not be unexpected for a couple who has worked through problems at one stage of life to have prior problems return or different ones develop as time passes, events occur, and new circumstances arise. For this reason, most contemporary couple therapies include some specific interventions prior to termination aimed at dealing with issues that may arise in the future. However, despite their obvious intuitive appeal, the efficacy of those interventions in forestalling or reducing future deterioration or divorce remains unknown.

### Client values

Couples exist within a broader socioecological as well as historical context. So, too, do the various models of couple therapy intended to treat couple distress and promote individual and relationship well‐being. That said, the contexts in which various couple‐based interventions were developed, and in which couple therapists are trained, may not mirror the diverse and emerging contexts shaping the set of values that each partner brings to therapy. How can couple therapists conduct effective therapy in a world in which values differ so mightily within and across couples?

For example, what processes are seen as essential for successful relationships? How much closeness or distance is viewed as optimal or acceptable? What to do when one aspect of relational life is problematic whereas others are satisfactory? How much to strive for what Finkel (2017) describes as the “all or nothing marriage” in which relationships are seen as needing to meet all individual needs? At what point is divorce viewed as a well‐considered option? How much might expectations for successful relationships vary with cultural context? At what point does good therapy entail challenging cultural expectations around such issues as gender inequality and relational violence?

Doherty (2022) and Lebow (2014) have written extensively about the crucial role of client and therapist values in couple therapy and about the complex and often unarticulated ways in which therapist values influence practice. LGBTQ therapists and those from various cultural contexts have added diverse vantage points to such discussion (Addison & Coolhart, 2015; Kelly et al., 2019). Couple therapy and, more importantly, couple therapists must remain aware, flexible, and responsive to the ways that values impact therapy—most especially in a world in which both conceptual models and related interventions are applied across diverse populations and cultures with dramatically different core beliefs and customs.

### Pandemic and postpandemic life

It is difficult to specify precisely how the Covid‐19 pandemic has affected couples and couple therapy beyond such simple observations as the increased use of teletherapy. Yet, there clearly have been profound effects (Stanley & Markman, 2020). Many of the standard interventions have needed to be adapted in response to dramatic increases in levels of both individual and relational stress and constraints driven by the pandemic. Although reports regarding couple satisfaction and divorce rates during the first 2 years of the pandemic are mixed, there is no doubt that for vulnerable couples both coping strategies and outside resources became more restricted and less sufficient. This necessitated an expanded vision of couple therapy during the pandemic and its aftermath. The conceptual scheme may remain largely the same—the therapeutic palette adapted to the times, but couple therapy is adapting. Specifically, observation suggests that themes once identified with existential therapy seem to be on the rise as they have in other turbulent times (Fraenkel & Cho, 2020).

### Inclusion in healthcare coverage

Couple therapy has succeeded in becoming widely disseminated as the preferred treatment for those encountering relational difficulties in the United States and much of the world. This accomplishment is especially remarkable given that there is little attention paid to couple therapy in most insurance and healthcare systems. For example, there is presently no separate Current Procedural Terminology (CPT) code for couple therapy (leaving the service coded as “family therapy”). Better procedures for coding couple therapy and couple relationship problems are sorely needed in healthcare systems, as well as a formal recognition of the cost‐effectiveness and therapeutic benefits of couple therapy for a broad spectrum of individual physical and mental health concerns of both partners and their offspring (Bradbury & Bodenmann, 2020; Ruddy & McDaniel, 2015). One estimate found couple therapy to be cost‐effective when paid for by the government to reduce public costs of divorce or when reimbursed by insurers to offset the increased healthcare expenses associated with divorce (Caldwell et al., 2007). Further arguments in favor of healthcare coverage for couple therapy include direct medical cost offsets and the fact that insurance companies already find it cost‐effective to reimburse for the prevention of other health and psychological problems (Clawson et al., 2018).

## CONCLUDING COMMENTS

This is an exciting time in the evolution of couple therapy! Collectively, there is remarkable depth and variety in today's approaches to couple therapy. Numerous approaches offer integration of evidence‐informed principles with clinical wisdom in the best of the scientist‐practitioner tradition. With an increasing foundation in relational science and evidence for their efficacy, such approaches continue to mature in their development. There is both a diversity in the most prominent approaches, but also an emerging and shared understanding of couple processes and core principles underlying couple‐based interventions. Both established clinicians and those in training may benefit from expanding their own theoretical lenses to examine the relative strengths, as well as limitations, of respective approaches to allow their own clinical repertoire to evolve as well—enhancing their skill sets for addressing the complexities of couples' challenges in a potentially more differentiated and effective manner.

## Footnotes

^1^
It is important to note here that ultimately the relative impact of in‐person vs. tele‐couple‐therapy is an empirical question that will require multiple studies to assess.

^2^
The historical trend to focus on white middle class couples is reflected in studies of clients in couple therapy research (Tseng et al., 2021).
